# The role of economic conditions and sustainable rural development on the sustainability of tourism development: evidence from China

**DOI:** 10.1007/s11356-022-24062-w

**Published:** 2022-11-28

**Authors:** Chenlei Xue, Yu-Te Tu, Mohammed Ananzeh, Ahmad Ibrahim Aljumah, Lam Minh Trung, Thanh Quang Ngo

**Affiliations:** 1 School of Civil Aviation, Xi’an Aeronautical Institute, Xi’an 710077 Shaanxi, China; 2 Department of Business Administration, Asia University, 500, Lioufeng Rd., Wufeng Taichung, 41354 Taiwan; 3 Management Department, Liwa College of Technology, Abu Dhabi, United Arab Emirates; 4 College of Communication and Media, Al Ain university, Abu Dhabi, United Arab Emirates; 5 Faculty of Business Administration, Van Lang University, 69/68 Dang Thuy Tram Street, Ward 13, Ho Chi Minh City, Binh Thanh District Vietnam; 6 School of Government, University of Economics Ho Chi Minh City (UEH), Ho Chi Minh City, 72407 Vietnam; 7 Research Group Public Governance and Developmental Issues, University of Economics Ho Chi Minh City (UEH), Ho Chi Minh City, 72407 Vietnam

**Keywords:** Tourism development, Gross domestic product, Inflation, Foreign direct investment, Exchange rate, Energy use, Sustainable rural development, Gross savings

## Abstract

At present, tourism is counted among those industries which have gained global attention due to rapid growth. Hence, a constant diversification in terms of destination is needed in tourism development. The recent trends of industry highlight the demand of alternative tourism types, among which nature-related tourism appears to be an emerging concept. In this regard, the present article investigates the impact of economic conditions and sustainable rural development on the sustainability of tourism development in China. The current research has gathered secondary data from the World Bank from 1981 to 2020. The quartile autoregressive distributed lag model has been applied to test the association between the variables. The results revealed that GDP, inflation, FDI, exchange rate, energy use, gross savings, and sustainable rural development have a significant and positive association with the sustainability of tourism development in China. Findings offer managerial implications recommending the local government to focus on the sustainability of rural development and economic conditions that may lead to the implementation of tourism-related development projects. This study also guides the policymakers in establishing policies related to tourism development using different economic conditions and sustainable rural development.

## Introduction

Over the past few decades, the importance of tourism is accelerating at a rapid pace. The importance of tourism is recognized by a number of scholars (Abdul Hamid et al. 2020; Arikan and Ünsever 2020; Pencarelli 2020). Economic growth of any country is associated with number of sectors. Out of those numbers of sectors, there are few sectors which are more important being associated with other sections of the economy. One of those sectors is tourism. Like tourism sector, it results in providing the jobs, bringing foreign capital in the country, supporting transportation industry, etc. The sustainability of tourism growth will strengthen the country’s economy. Keeping in view the increasing importance of the sustainability of the tourism industry for the economy, a number of scholars have explored the sustainable tourism development and economy nexus and proposed that there is a strong association between sustainable tourism development and economy (Ainou et al. 2022; Danish and Wang 2018; Pulido-Fernández and Cárdenas-García 2020). The travel and tourism business is an important economic sector for both developed and emerging nations since it creates jobs and increases revenue. Indeed, sustainable tourism is a major source of revenue for many of these countries. This sector is also critical for economic development, and if the sustainable tourism sector contracts in the future, these economies will suffer. To further expand the tourism sector (with the aim of bringing sustainability), various resources are required, and various economic factors have been identified as being critical in this regard like gross domestic product (GDP), foreign direct investment, inflation, exchange rate, energy use, gross savings, and sustainable rural development. Indeed, sustainable tourism remains an activity in which capital, infrastructure, knowledge, and access to global marketing and distribution chains are critical, and economic factors such as foreign direct investment (FDI) and exchange rate are regarded as some of the most effective engines for supporting such critical elements. Such economic factors like foreign direct investment (FDI), exchange rate, inflation, energy, and economic growth do impact the tourism sector growth in the country. Due to the importance of this sector for the economy, the present study selected the tourism sector.

Further to the argument, the development of tourism in villages could enhance economic growth which ultimately is the reason of job creation and quality life; however, on the other hand, there exists plenty of drawbacks such as environmental damage, local resources depletion, and infrastructure overloading (Ali et al. 2022). Due to this fact, it is imperative to evaluate the relationship of sustainable tourism development and the promotion of sustainable development in rural areas. Similarly, another pile of literature argued that sustainable development could only be attained when there is a balance between economic growth and environment. In this regard, the exploitation and protection of villages must be balanced during touristification. Researches widely argued that sustainable tourism should not be focused on ecological environment only but also considers social order and economic function as well as these are equally important aspect of sustainable rural tourism (Bai et al. 2022; Firman et al. 2022; Li et al. 2019).

**Fig. 1:**
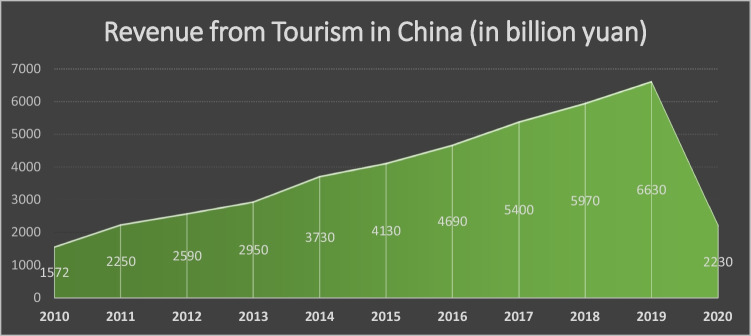
Revenue from tourism in China (WDI)

China is a country with a strong history. Additionally, China has a very rich culture. This strong history and culture attract the world towards China to explore its culture as well as history. Another factor that attracts tourists to China is its rapid development in the economy. People from around the globe prefer to select China for employment. The sustainability in the Chinese tourism sector will support its economy at a high level. China has a long tourism history (Chien 2022a; Su et al. 2018). Since the onset of reform and opening in the early 1980s, tourism has been an important contributor to China’s domestic economy. This travel boom is being fueled by the rise of an affluent middle class and the loosening of restrictions on locals and international travelers alike. The Chinese tourism sector has become one of the most closely studied inbound and outbound tourist marketplaces in the world. In 2019, the number of domestic travels in China surpassed six billion, suggesting a ten-fold increase in comparison to 10 years previously. As of 2019, the total income generated by China’s travel and tourism business was over 5.7 trillion yuan, reflecting a steady increase over the previous decade. By 2028, the industry was predicted to contribute directly 3.3% to China’s gross domestic product (GDP). To carry this momentum, there is an urgent need of sustainable development in tourism sector. Meanwhile, the tourist business employed almost 28 million people. When indirect employment is taken into account, the influence of tourism on China’s labor market is considerably greater. China has many attractions, including historical monuments and artefacts, economic hotspots, and a culturally diversified population of minorities. As a result, numerous countries, including Thailand, Japan, South Korea, the Maldives, Russia, and the UK, have made the country one of their most popular tourism destinations. In 2019, China’s 145 million incoming tourists generated roughly 36 billion dollars in income. The bulk of incoming visitors landed in Guangdong Province, which is next to Hong Kong SAR in the south. In terms of outbound tourism, Chinese outbound passengers spent around 254 billion dollars. With a share of 61%, Europe was the most popular vacation destination among young Chinese luxury tourists, followed by Japan and South Korea. Revenue generated from tourism in China is given in Fig. 1. Moreover, in China, many of the traditional villages have come to the point where they were able to achieve rural revitalization by promoting the notion of comprehensive development which says “production, life, and ecology.” Also, villages nowadays are no longer ordinary villages; in fact, they have gained market significance due to becoming the tourism region. Hence, we can say that rural sustainable development is the driving force of sustainable tourism development as it simultaneously displays the combination of multiple activities such as agricultural, ecological, and traditional that are sufficient enough to attract urban residents (Chien et al. 2022b; Gao et al. 2019)

In this regard, the present article attempts to address several concerns which according to author’s knowledge have been neglected in prior evidences. Sustainable tourism development along with sustainable rural development is considered to be one of those contemporary concerns which have caught enough attention from scholars; however, one cannot claim that the said areas have been explored fully especially with certain economic conditions. Triantafillidou and Tsiaras (2018) surely investigated the nexus between entrepreneurship, innovation, and sustainable tourism development; however, in contrast, our study focuses on the relationship of economic condition and sustainable rural development with sustainable tourism growth in the context of China. Similarly, Movono et al. (2018) tested sustainable tourism development relationship with ecological and economic factors in the context of Fiji, whereas the present model tested the model in Chinese perspective by removing ecological factors. Furthermore, Pratama and Mandaasari (2020) tested the sustainable tourism development from cultural perspective, whereas the proposed model examined it from economic conditions’ point of view. Hence, several contributions can be drawn from this article. It highlights the significance of economic conditions for sustainable tourism development in the economy of China. Secondly, it helps the researchers to identify the economic conditions like economic growth, FDI, inflation, exchange rate, energy use, sustainable rural development, and gross saving importance for any country tourism.

The study is structured in five sections. Introduction is the first section where the issue has been highlighted and linked with country context. The second section covers the literature review part where past literature has been discussed and gaps are highlighted to enhance the significance of study. Methodology section covers the data technique and measurement of the variable. The next section includes the discussion part where results are contrasted in the light of past studies. Lastly, recommendations, implications, and limitations have been discussed under conclusion part.

## Literature review

The FDI is one of the prime factors which regulate country’s economy. When a firm, sector, individual, or entity is controlled from outside, its entity or country is narrated as a foreign direct investment (FDI). Usually, the FDI is practiced in open economies where there is an abundance of skilled labor as well as cheap resources. The FDI usually flows from developed to developing countries with the intention to have cheap resources and increase profitability. The FDI is considered one of the major pillars of the economy. Studies proposed that the FDI directly impacts the economy of any country (Chien et al. 2022c; Nunkoo and Seetanah 2018; Sheng et al. 2021). In this context, Fauzel (2021) explored the FDI nexus with tourism in small island economies. The data set of 23 years ranging from 1995 to 2018 was tested to check both the long as well as the short-run impact of FDI. The results of the study revealed a positive association of FDI with tourism development in the selected 17 economies. Further, FDI is the core element towards tourism development. Similarly, Amin et al. (2020) also explored the FDI impact from Bangladesh’s perspective. The time-series data set from 1972 to 2017 was tested by applying multiple approaches like ARDL and DOLS. The results revealed a causality between FDI and tourism development in Bangladesh and further suggested the policymakers to formulate a sustainable tourism policy. Africa is considered one of the important regions for tourism. In this context, Osinubi et al. (2022) tested how FDI impact tourism. The study used the 10 most preferred African countries’ panel data of 24 years from 1995 to 2019. The results proposed that FDI significantly and positively affect tourism development in selected African economies.

From the past few decades, the tourism sector has witnessed rapid growth all around the globe. The contribution of this sector to the economy makes it more important for the country. Another element which enhances its importance is its association with the number of other sectors like labor, traveling, hospitality, and taxation. The studies proposed that there is a strong association between inflation and tourism development in the country (Chien 2022d; Pektas and Unluonen 2020; Shaari et al. 2018). In this context, Meo et al. (2018) explored the nexus between inflation and tourism development in Pakistan. The data set of 35 years from 1980 to 2015 was tested by employing the ARDL approach. The results revealed that there is a significant positive association between inflation and tourism development in Pakistan. Except for inflation, there are a number of other factors like security condition and socioeconomic and financial condition which impact the tourism development in the country. Thus, Athari et al. (2021) explored the inflation nexus in 76 destinations. The panel data of 23 years from 1995 to 2917 was tested by employing the GMM approach. The results of the study revealed that there is an association between inflation and tourism development in the selected destinations. Europe is considered one of the tourism hubs in the globe, as Europe is a combination of multiple cultures which attracts international tourists. In this context: Yong (2014) explored the inflation and tourism development relation in 14 European countries. The data set of 22 years from 1988 to 2010 was tested by employing the FGLS method. The results revealed that there is an association between inflation and tourism in the selected European economies. The tourism and inflation nexus is not only important for developing but also for developing countries. There are multiple forms of tourism like a geological truism and religious tourism. It also noted that religious tourism also plays a vital role in the country’s economy. In this context, Achyar and Hakim (2021) also tested the inflation, tourism, and economic growth nexus in Indonesia. The data set from 1990 to 2019 was tested, and the results revealed that there is an association between inflation and tourism development in Indonesia in the long run.

The tourist industry is regarded to positively contribute to a country’s economic growth process through several channels, including the fact that it is a currency earning sector, that supports physical and human capital accumulation and those pushes (and employs) technology and innovation. At the same time, tourism stimulates other economic sectors, such as transportation, hospitality, and shopping, both directly and indirectly (Gao et al. 2021; Haroon et al. 2021; Pulido-Fernández and Cárdenas-García 2020). International tourism, in particular, is a source of foreign money that supports the procurement of capital goods and technology that may be employed in other manufacturing processes. Furthermore, it plays an important role in supporting investments in new infrastructure and increasing competitiveness, hence providing jobs and corresponding household income. Finally, it should be highlighted that tourism is an important industry for the dissemination of technological information, and it has the ability to drive research and development. Many governments are focusing more on supporting and promoting tourism as a possible source of growth and jobs, as well as a sector that adds value to cultural, natural, and other capital that has no market price (Liu et al. 2022b; Sadiq et al. 2022a). In this context, Brida et al. (2020) explored the economic growth and tourism development nexus. The data set of 80 countries covering the tenure from 1995 to 2016 was tested by employing the symbolic time-series technique. The data of low- and high-tourism countries were tested at the same time. The results revealed that there is causality between economic growth and tourism development in the selected countries. Further, Danish and Wang (2018) also explored the nexus between economic growth and tourism in BRICS. The data selected for the study range from 1995 to 2014. The results revealed that tourism significantly supports economic growth. The study also recommended that policymakers formulate sustainable tourism policies for betterment. Additionally, Liulov et al. (2020) also explored this nexus in 6 economies in the presence of COVID-19. The change in tourism trend was also tested in the study. The data set of 6 months was tested. The results revealed that in the presence of COVID-19, the change in truism trends significantly impact the economic growth of the country.

With the passage of time, the importance of tourism is accelerating at a rapid pace. One of the reasons is that it is associated with a number of other factors like the hospitality industry, investment attraction, employment, and banking sector. The exchange rate significantly impacts tourism development in developed as well as developing countries. The present study aimed to test the exchange rate and tourism development factors. In this context, Akadiri and Akadiri (2021) explored the economic growth and tourism development nexus by adding exchange rate variables. The data of 16 island states from 1995 to 2016 was tested by employing different approaches. The results revealed that the exchange rate impacts both economic growth as well as tourism in the selected islands. Similarly, Munir and Iftikhar (2021) also explored the tourism, foreign direct investment, and exchange rate relationship in different South Asian countries. The data of Bangladesh, India, Nepal, and Sri Lanka as well as Pakistan from 1995 to 2019 was tested by employing ARDL and NARDL approaches. The results proposed that the increase in the exchange rate decreases tourism. The impact of exchange rates on trade balances, particularly the current account, has long been a source of concern for academics and policymakers alike. Thus, Işik et al. (2019) explored the nexus between the exchange rate and tourism development in Spain. The results revealed that the exchange rate has a positive association with tourism in Spain. In the same way, Sharma and Pal (2019) tested the relationship between exchange rate volatility and demand for tourism in India. The data from 2006 to 2008 was tested by employing the ADL model. The results revealed that the exchange rate volatility does impact the tourism demand in India.

Energy is considered one of the basic contributors to the economy. Over the past few decades, the demand and usage of energy have accelerated at a rapid pace. The present study aimed to check the energy use impact on tourism development. Liu et al. (2019) explored the relationship between energy and tourism from a Pakistani perspective. The data selected for the study was from 1980 to 2016. The data was tested by employing the ARDL model. The results revealed the use of energy strongly influenced by the tourism development in Pakistan, as tourism helps an economy’s growth by providing foreign currency and job opportunities. Tourism, on the other hand, leads to increased energy consumption due to numerous visitor activities such as hotel rooms and transportation. Thus, Khanal et al. (2021), also explored the energy and tourism relationship nexus from the Australian perspective. The analysis used data from the Australian Bureau of Statistics, the BP Statistical Review, and the World Development Indicators to span the previous 4 decades, i.e., from 1976 to 2018. To evaluate the association, enhanced Dickey-Fuller, Phillips-Perron, autoregressive distributed lag (ARDL) bound tests, Johansen and Juselius, Bayer-Hanck co-integration test, and numerous important diagnostic tests were used. According to the projected results, tourist arrivals, GDP, and financial growth have a considerable long-run co-integrating relationship with energy consumption. Based on the study’s findings, policy recommendations are made. The literature also supports the relationship between energy and tourism (Dogru et al. 2020; Gulistan et al. 2020; Zhao et al. 2021, 2022).

China is a country that is rich in culture, history, etc. Annually there is a number of individuals from all around the globe visiting China for different purposes, i.e., business and study. The industry which gets the most effective when individuals visit China is the tourism industry. On the other hand, being a large country area, China is a combination of urban and rural areas. The tourist usually prefers urban areas by ignoring rural due to less information. Here tourism can play a vital role to promote the rural economy. There is a positive association between rural development and tourism (Nguyen et al. 2021b; Sadiq et al. 2022b, c; Tan et al. 2021). In this context, Nooripoor et al. (2021) investigated whether tourism plays any role in rural development in Iran. As Iran is also a country that has a rich history, but there is a need to promote the rural areas in order to support the economy as well as society. The data of 150 households belonging to rural areas were collected and tested. The findings of the study proposed that tourism plays a vital role in sustainable rural development in Iran. There are different forms of tourism like rural, urban, and astro-tourism. South African land is full of rural areas. There is a need to promote this rural section through different means like tourism. In this context, Jacobs et al. (2020) investigated whether astro-tourism plays any role in rural development in South Africa. The results of the study proposed that astro-tourism plays a vital role in rural areas’ development in Africa. Similarly, Atun et al. (2019) investigated whether rural development results in sustainability with the help of tourism. The study was conducted in Cyprus. By providing an overall framework for tourism and rural development, the study proposed that tourism can help to promote rural development in a country like Cyprus. Furthermore, Panzera and Postiglione (2020) did a systematic review of rural development. The data of articles ranging from 2009 to 2019 were selected and reviewed in the investigation. The findings proposed that there is a number of factors which have positive effect on sustainability of rural development and tourism in one of the core factor from those (Fernández Martínez et al. 2020; Woyesa and Kumar 2021). Thus, it is hereby proposed that there is a close association between tourism development and rural development sustainability.

The culture of any country strongly influences the people who live in it. The culture also affects the living standard as well as habits of the people. Another factor which strongly influences the way of living of any country people is the economic conditions like inflation and employment. As there is a direct association between a country’s economic condition and people’s way of living (Kamarudin et al. 2021; Samarskaya et al. 2019; Schirripa Spagnolo et al. 2018), thus, the saving of any country people depends upon the economic conditions. A country with healthy economic conditions like better job opportunities will help the people to save their money to fulfill their plans like higher education, better food, and tourism. Thus, there is a linkage between country people’s economic conditions and tourism (Khattak et al. 2021; Kurnianingsih et al. 2021). If people have healthy savings in their pockets, only then they can explore their own country as well as other countries by visiting them. A country with low income results in less involvement in tourism activities. Another factor that impacts the tourism activities in the country is the spending habit of the people. The economic conditions of any country also influence the spending habits of the country’s people. If the country’s economic conditions are good, like there is an abundance of jobs, only then they can save from their income and spend the same for their hobbies like tourism. Thus, the economic conditions influence people’s spending habits (Kumai et al. 2022; Lan et al. 2022; Liu et al. 2022a; Chien et al. 2021; Yaşar et al. 2019). The more the people of any country will be able to save from their income after paying for their regular expenses, the more the chances they will prefer to go for tourism. The people with less income are not in the position to go for tourism due to less or no saving after spending for their monthly or regular expenses. In this context: Winter and Kim (2021) explored the relationship between poverty and tourism in Brazil. The results of the study revealed that there is an association between poverty and tourism. The more the poverty is, the less the tourism activities will be. Further, Lertputtarak and Deeprom (2021) explored tourism management in low-income areas in Thailand. The results of the study revealed that there is an association between income and tourism promotion in Thailand. Thus, it is hereby concluded that economic condition like employment influences the people’s saving capacity and the saving capacity of the people further impacts the tourism activities in the country.

## Methods and material

The research investigates the impact of economic conditions such as GDP, inflation, FDI, exchange rate, energy use and gross savings, and sustainable rural development on the sustainability of tourism development in the context of China. The current research has gathered secondary data from the World Bank from 1981 to 2020. The study equation is given below:

1 $${STD}_t={\alpha}_0+{\beta}_1{GDP}_t+{\beta}_2{FDI}_t+{\beta}_3{INF}_t+{\beta}_4{ER}_t+{\beta}_5{EU}_t+{\beta}_6{GS}_t+{\beta}_7{SRD}_t+{e}_t$$

where

STD: sustainability of tourism development
*t*: time period
GDP: gross domestic product
FDI: foreign direct investment
INF: inflation
ER: exchange rate
EU: energy use
GS: gross savings
SRD: sustainable rural development

Table 1 illustrates the complete details of variable sources.

**Table 1:** Variables with measurements

Variables	Measurement	Sources
Tourism development	International tourism, receipts (% of total exports)	Alola et al. (2019)
Economic growth	GDP growth (annual %)	Jakovljevic et al. (2020)
Foreign direct investment	FDI net inflows (% of GDP)	Nguyen et al. (2021a)
Inflation	Consumer prices (annual %)	Usman and Musa (2018)
Exchange rate	Real effective exchange rate index	Angelo (2021)
Energy use	Energy use (kg of oil equivalent per capita)	Saint Akadiri et al. (2019)
Gross savings	Gross savings (% of GDP)	Makori et al. (2022)
Sustainable rural development	Rural population growth (annual %)	Zhang et al. (2022)

The current study has run the descriptive statistics in order find the normality of data. Moreover, correlation matrix was also performed to identify the directional linkage between the variables. Also, the stationarity of the constructs has been checked via Augmented Dickey-Fuller (ADF) test. The equation for the ADF test is given as under:

2 $$d\left({Y}_t\right)={\alpha}_0+\beta t+{YY}_{t-1}+d\left({Y}_t\left(-1\right)\right)+\kern0.5em {\upvarepsilon}_t$$

If the ADF test results show all the constructs are stationary at the level, then the vector error correction model (VECM) is appropriate, but if some variables are stationary at the level and some are at the first difference, then the ARDL model is appropriate.

The present article has applied the QARDL model to explore the linkage between the constructs. It is a suitable model when the constructs are stationary at the level and first difference. In addition, it is also the best model even small sample size used by the study, as the current study has used only 40 observations. Moreover, QARDL has also adjusted the adverse effects of autocorrelation and heteroscedasticity that normally exist in the model. Firstly, the ARDL equation is given below:

3 $$\Delta {STD}_t={\alpha}_0+\sum {\delta}_1\Delta {STD}_{t-1}+\sum {\delta}_2\Delta {GDP}_{t-1}+\sum {\delta}_3\Delta {FDI}_{t-1}+\sum {\delta}_4\Delta {INF}_{t-1}+\sum {\delta}_5\Delta {ER}_{t-1}+\sum {\delta}_6\Delta {EU}_{t-1}+\sum {\delta}_7\Delta {GS}_{t-1}+\sum {\delta}_8\Delta {SRD}_{t-1}+{\varphi}_1{STD}_{t-1}+{\varphi}_2{GDP}_{t-1}+{\varphi}_3{FDI}_{t-1}+{\varphi}_4{INF}_{t-1}+{\varphi}_5{ER}_{t-1}+{\varphi}_6{EU}_{t-1}+{\varphi}_7{GS}_{t-1}+{\varphi}_8{SRD}_{t-1}+\kern0.5em {\upvarepsilon}_t$$

The present article has applied the QARDL model because the aim of the study is to check the quartile impact of the variables. The current study has taken the quartile such as *τ* = {0.1, 0.2, 0.3, 0.4, 0.5, 0.6, 0.7, 0.8, 0.9}. The equation of QARDL is given below:

4 $${Q}_{STD t}=\alpha {\left(\tau \right)}_0+\sum \nolimits_{i=1}^{n1}{b}_i\left(\tau \right){STD}_{t-i}+\sum \nolimits_{i=0}^{n2}{c}_i\left(\tau \right){GDP}_{t-i}+\sum \nolimits_{i=0}^{n3}{d}_i\left(\tau \right){FDI}_{t-i}+\sum \nolimits_{i=0}^{n4}{e}_i\left(\tau \right){INF}_{t-i}+\sum \nolimits_{i=0}^{n5}{f}_i\left(\tau \right){ER}_{t-i}+\sum \nolimits_{i=0}^{n6}{f}_i\left(\tau \right){EU}_{t-i}+\sum \nolimits_{i=0}^{n7}{g}_i\left(\tau \right){GS}_{t-i}+\sum \nolimits_{i=0}^{n8}{g}_i\left(\tau \right){SRD}_{t-i}+{\varphi}_1\left(\tau \right){STD}_{t-1}+{\varphi}_2\left(\tau \right){GDP}_{t-1}+{\varphi}_3\left(\tau \right){FDI}_{t-1}+{\varphi}_4\left(\tau \right){INF}_{t-1}+{\varphi}_5\left(\tau \right){ER}_{t-1}+{\varphi}_6\left(\tau \right){EU}_{t-1}+{\varphi}_7\left(\tau \right){GS}_{t-1}+{\varphi}_8\left(\tau \right){SRD}_{t-1}+{\upvarepsilon}_t$$

In the above equation, *τ* shows quartile, *δ* shows long run associations, and *φ* shows the short-run linkage. Finally, the current article also checks the model stability through CUSUM and CUSUM square.

## Study results

The results from Table 2 exposed that the highest tourism development was 10.452% in 2020, while the lowest STD was 2.221% in 1989. In addition, the results show the that highest GDP growth was 14.225% in 2017, while the lowest GDP growth was 2.348% in 1981. Moreover, the results exposed that the highest FDI was 6.187% in 2016, while the lowest FDI was 0.135% in 1998. In addition, the results show that the highest INF was 18.812% in 1985, while the lowest INF was −1.401% in 1999. Furthermore, the results exposed that the highest ER was 202.337% in 1989, while the lowest ER was 70.525% in 2015. In addition, the results show that the highest EU was 1268.133 in 1983, while the lowest EU was 243.375 in 2020. Moreover, the results exposed that the highest GS was 51.788% in 2011, while the lowest GS was 33.150% in 1996. Finally, the results exposed that the highest SRD was 0.684% in 1987, while the lowest SRD was −2.636% in 2020. Table 2 shows year wise descriptive statistics.

**Table 2:** Descriptive statistics (years)

Years	STD	GDP	FDI	INF	ER	EU	GS	SRD
1981	5.379	2.348	1.443	2.419	123.382	982.603	44.533	0.334
1982	4.984	5.950	1.311	2.899	120.888	963.648	43.773	0.486
1983	4.589	10.114	3.484	3.825	85.846	1268.133	45.672	0.629
1984	4.195	10.038	3.487	1.128	88.376	1118.432	42.413	0.470
1985	3.800	11.223	1.023	18.812	117.170	720.341	37.979	0.495
1986	3.405	11.657	0.848	7.234	107.179	694.422	37.234	0.595
1987	3.011	8.950	0.623	12.964	124.07	671.210	35.250	0.684
1988	2.616	13.431	0.536	14.939	171.750	657.760	35.279	0.662
1989	2.221	15.192	0.484	16.913	202.337	651.075	34.896	0.559
1990	9.113	8.490	3.475	0.348	92.765	898.987	35.740	0.465
1991	9.112	8.336	3.513	0.719	96.764	928.811	37.269	0.175
1992	8.780	9.134	3.609	−0.732	94.552	984.811	38.883	−0.004
1993	8.669	6.750	1.694	2.075	121.739	755.148	44.486	−0.116
1994	8.503	6.947	1.349	1.593	120.094	736.193	44.905	−0.177
1995	8.336	6.849	1.556	2.000	123.670	717.239	44.381	−0.262
1996	8.170	10.770	0.276	2.369	227.008	698.284	33.150	−0.345
1997	8.004	9.017	0.210	2.761	230.937	679.330	33.803	−0.407
1998	7.837	5.113	0.135	3.152	242.070	660.375	39.819	−0.517
1999	10.012	7.662	3.749	−1.401	92.687	641.421	36.558	−0.655
2000	9.358	7.846	4.436	−0.773	98.016	622.466	38.347	−0.778
2001	8.984	9.237	4.725	2.786	93.004	603.511	39.742	−1.188
2002	9.083	9.923	4.652	8.313	86.351	584.557	38.759	−1.470
2003	9.159	10.954	4.880	16.791	78.598	565.602	39.530	−1.596
2004	9.235	9.551	3.709	5.554	102.696	546.648	49.233	−1.704
2005	9.312	7.864	2.827	2.620	108.677	527.693	48.665	−1.781
2006	9.388	7.766	3.040	2.621	114.617	508.739	47.378	−1.811
2007	9.464	7.426	2.559	1.922	118.314	489.784	47.616	−1.877
2008	9.540	7.041	2.192	1.437	129.970	470.829	45.418	−1.963
2009	9.616	10.636	4.004	3.175	100.000	451.875	51.327	−2.043
2010	9.692	9.399	2.569	−0.728	101.105	432.920	50.373	−2.133
2011	9.768	9.651	3.734	5.925	97.011	413.966	51.788	−2.017
2012	9.844	14.231	4.401	4.817	89.329	395.011	50.275	−1.888
2013	9.920	12.721	4.509	1.649	86.255	376.057	48.261	−1.955
2014	9.996	11.395	4.554	1.776	84.916	357.102	45.955	−2.057
2015	10.072	13.037	5.987	24.257	70.525	338.147	41.697	−2.169
2016	10.148	13.884	6.187	14.610	89.961	319.193	41.707	−2.244
2017	10.224	14.225	2.613	6.354	84.547	300.238	40.606	−2.265
2018	10.300	9.263	1.139	3.557	88.301	281.284	38.501	−2.409
2019	10.376	3.920	0.966	3.052	100.537	262.329	36.694	−2.516
2020	10.452	4.206	0.976	18.246	136.309	243.375	35.877	−2.636

In addition, from descriptives, minimum and maximum values along with average values have been extracted. From Table 3, we can observe that STD has 8.117% average value followed by GDP growth 9.3%, FDI 2.687%, INF 5.549%, ER 116.058%, EU 612.989%, GS 41.8445, and SRD −0.936%.

**Table 3:** Descriptive statistics

Variable	Obs	Mean	Std. dev.	Min	Max
STD	40	8.117	2.486	2.221	10.452
GDP	40	9.304	2.947	2.348	15.192
FDI	40	2.687	1.693	0.135	6.187
INF	40	5.549	6.406	−1.401	24.257
ER	40	116.058	41.764	70.525	242.07
EU	40	612.989	243.247	243.375	1268.133
GS	40	41.844	5.392	33.150	51.788
SRD	40	−0.936	1.143	−2.636	0.684

From Table 4, we can observe that all the values lies between −1 and 1; hence, it shows that GDP, inflation, FDI, exchange rate, energy use, gross savings, and SRD have a positive association with the sustainability of tourism development in China. The correlation matrix is given in Table 4.

**Table 4:**
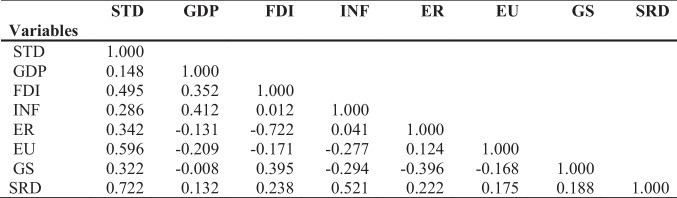
Correlations matrix

Furthermore, the current article also checks the stationarity of the constructs using the ADF test to apply the appropriate model in the study. The results indicated that STD, GDP, INF, and SRD are stationary at a level, while FDI, ER, EU, and GS are stationary at first difference. ADF test results are given in Table 5.

**Table 5:** Unit root test

Augmented Dickey-Fuller Test (ADF)	Level	t-statistics	*p*-values
STD	I(0)	−2.653	0.021
GDP	I(0)	−2.172	0.037
FDI	I(1)	−5.982	0.000
INF	I(0)	−2.621	0.017
ER	I(1)	6.192	0.000
EU	I(1)	−4.829	0.000
GS	I(1)	−5.922	0.000
SRD	I(0)	−2.162	0.039

The results revealed that GDP, inflation, FDI, exchange rate, energy use, and gross savings are positively correlated with tourism development in China. The results also exposed that GDP has a significant association with STD in quartiles 1 to 5 and 7 to 8 in the short run and quartiles 1 to 5 and 8 to 9 in the long run. In FDI, it also shares positive connection with STD in quartiles 1 and 2 to 7 and quartiles 1 to 2, 3 to 4, and 8 to 9 in the short and long run, respectively. Furthermore, INF and STD are significant to each other in quartiles 1 to 6 and 9 and quartiles 1 to 4 and 7 to 9 in the short as well as the long run. Moreover, the ER also according to results is significantly associated with STD in quartiles 1 to 4, 6, and 8 to 9 in the short run and quartiles 1 to 4 and 7 to 8 in the long run. The results also exposed that the EU and STD are significant in quartiles 1 to 6 and 8 in the short run and quartiles 1 to 5 and 7 and 9 in the long run. Moreover, according to findings, GS and STD are significant to each other in quartiles 1 to 4 and 7 to 9 in the short run and quartiles 1 to 4, 6, and 8 to 9 in the long run. Finally, in case of SRD, it proved its significance with STD in quartiles 1 to 8 in the short run and quartiles 1 to 7 in the long run. The QARDL results are given in Table 6.

**Table 6:** QARDL model

Panel A: short-run coefficients									
Variables	Q0.1	Q0.2	Q0.3	Q0.4	Q0.5	Q0.6	Q0.7	Q0.8	Q0.9
GDP	0.76**	0.65**	0.72***	0.32**	0.54*	0.23	0.65**	0.87*	0.10
FDI	0.54**	0.12	0.46**	0.51**	0.21*	0.91***	0.59*	0.13	0.22
INF	0.82**	0.22*	0.62**	0.51*	0.71*	0.96**	0.51	0.13	0.53**
ER	0.18*	0.32*	0.77**	0.92***	0.12	0.32*	0.02	0.27*	0.78**
EU	0.62**	0.51*	0.62*	0.79***	0.74*	0.62**	0.12	0.72*	0.22
GS	0.29*	0.82*	0.66*	0.53**	0.11	0.15	0.43*	0.62*	0.55*
SRD	0.28*	0.14*	0.36*	0.41*	0.22*	0.63*	0.18*	0.23*	0.04
Panel B: long-run coefficients									
Variables	Q0.1	Q0.2	Q0.3	Q0.4	Q0.5	Q0.6	Q0.7	Q0.8	Q0.9
GDP	0.54*	0.39*	0.71**	0.67***	0.51*	0.10	0.21	0.53*	0.59**
FDI	0.62**	0.52*	0.17	0.53**	0.42*	0.21	0.33	0.54*	0.76***
INF	0.41*	0.62**	0.22*	0.64**	0.18	0.10	0.51*	0.19*	0.91**
ER	0.54*	0.68**	0.29*	0.54**	0.21	0.10	0.55**	0.65**	0.12
EU	0.74*	0.32**	0.42*	0.89**	0.84**	0.14	0.73**	0.22	0.35*
GS	0.59**	0.72**	0.63**	0.42*	0.23	0.41*	0.11	0.71**	0.56**
SRD	0.34*	0.72**	0.27*	0.35*	0.62*	0.55*	0.34*	0.02	0.10
Panel C: diagnostics									
Ad. R square	0.46								
CUSUM	S								
CUSUMQ	S								

## Discussions

Findings reveal that economic growth is positively correlated to sustainable tourism development. The study results are consistent with the study of Inchausti-Sintes et al. (2021) and Moslehpour et al. (2022), which examines the role of economic growth in accelerating and sustaining tourism development, and according to them, when any country shows rapid progress, the financial strength of the government and individual business enterprises increases, especially those which are engaged in tourism activities. This enhances their power to execute the strategies and plans for the improvement in the tourism services like infrastructure, accommodation, recreation, and traveling, so that the tourists’ interest can be aroused and maintained. That is why the increase in economic growth leads the country to bring sustainable development in tourism. The evidence are consistent with Eyuboglu and Eyuboglu (2020), who emphasize the economic growth for getting high sustainability in tourism development. They proclaim that when there is an increasing economic growth rate and entirely new technologies or technologies with less or more improvements are being introduced, there is a great assistance in the performance of tourism services in a better way. So, the increased economic growth accelerates tourism development and is helpful to developing sustainability in tourism firms’ progress. Obtained results are in line with Gao et al. (2021), which posits that when there is high economic growth, the living standard of the people is high, and the local culture is richer in a variety of resources, events, and recreational places; so, the chances of developing sustainability in tourism development are greater.

The results have stated that FDI and sustainability of tourism development shares positive connection. These results agree with Fauzel (2020), which depicts that when there is a large amount of investment from the companies or individuals in the foreign, in the tourism firms operating in some domestic regions or which arrange for conducting international tours, the firms can bring technological improvements which all require the heavy investment. These technological improvements enhance the quality of information management, guidance and consultation, accommodation services, visiting, and restaurant services, leading to tourism development at a consistent rate. Hence, according to the authors’ views, an increase in foreign direct investment enables the tourism industry to make sustainable development. These results also match with Zhuang et al. (2021), which highlights that foreign direct investment has great significance in the development of a country’s tourism industry. The financial resources, as a result of the increased financial investment, makes it possible for the state to develop the tourism industry with the implementation of innovative technologies, energy-efficient logistics, extension to restaurants and accommodation, reliability of infrastructure, and improvement to recreation. So, by encouraging foreign direct investment, tourism development can be made sustainable. These results also relate to Sokhanvar (2019), a study on foreign direct investment that impacts on tourism development. The study reveals that in the tourism industry, having investment from both domestic and international sources, the firm management can perform different practices efficiently and give promotion to the industry within the country and help keep the tourism development sustainable.

The results have shown that sustainability of tourism development and inflation share positive connection. These results are in line with Athari et al. (2021), which indicates that during inflation, the prices of services along with the goods also rise within the country. The increase in the profits is earned by the tourism firms at the different platforms of tourism services. The increased profits enable the firms to bring development in their services through improvement in and extension to tourism facilities. The consistently high rate of profitability enables the tourism firms to contribute to sustainable tourism development. The findings show consistency with Meo et al. (2018), which analyzes the role of inflation in the sustainability of tourism development in a country. The study implies that when there is inflation within the country, the productivity level within the industrial and service sector is high. The large production of goods and services increases resources for the industrial firms which belong to a large number of businesses of different nature, like food processing, transportations, infrastructure, and construction. So, in inflation period of economy, the tourism industry can be led towards SD. Findings confine with Hang et al. (2020), according to which inflation is an indicator of TD. The study implies that during the inflation period, the economic activities are at a peak. The larger population is on employment, and the increased living standard allows them to pay for the creation and enjoyment through tourism. This leads to sustainable tourism development within the country.

Exchange rate also seems to share positive connection with sustainability of tourism development. Hence, it matches with the findings of Lee et al. (2021), which analyzes that when domestic tourism firms are engaged in serving the foreigners as well, for providing their creations and refreshment from the daily fatigue of life, the change in the exchange rate affects the earnings from the tourism. The future planning about the expansion of the tourism business depends upon the earnings of the tourism firms. Thus, the consistent increase in the exchange rate increases tourism development and make it sustainable. These results also agree with Usman et al. (2021), which reveals that in case the exchange rate is increasing, the value of the currency is getting high, and the earnings through the tourism packages, food and beverages, and shopping are greater in value. The increased earnings enable the tourism firms to bring value addition and variety addition to the tourism services provided by them. In this way, sustainability can be created in the tourism development. Evidences are in line with Shahbaz et al. (2019), which indicates that when there is an increasing exchange rate, just like exports, the tourism services sold to foreigners bring larger profits and opens the ways for the tourism firms to bring innovativeness in the tourism resources, parks, accommodation, traveling, and other accessories. The innovativeness in the tourism practices assists in creating sustainable tourism.

Moreover, the energy use and the sustainability of tourism development are also positively related. Hence, consistent with Khan et al. (2020), which shows that tourism is a bundle of practices, these all activities require energy for functioning. In the country where the energy resources are available in large amount and the tourism firms can utilize them easily, they can perform the business functions effectively, and even they can expand the scope of the tourism services. So, energy consumption positively contributes to STD. The findings are supported by Sghaier et al. (2019), which posits that the amount of energy to be consumed and its quality determine the impacts of the tourism activities on social progress and environmental quality. When the tourism firms utilize renewable energy resources instead of fossil fuels, it provides a clean and healthy environment to tourists, reduces their fear about their health protection, and encourages more tourists towards these tourists’ destinations. Hence, the effective energy consumption pattern leads to STD. The findings show consistency with Aslan et al. (2021), which highlights that the use of clean energy resources is not only favorable for the tourists, but it also assures the availability of natural tourism resources like food, greenery, parks, mountain, hills, waterfall, and wildlife which provides for recreation. So, energy use matters a lot in sustainable tourism development.

The results have shown that gross savings have a positive relation to sustainable tourism development. These results match with Gupta and Dutta (2018), which examines that the gross savings enhance the firms’ ability to make additional investments to the tourism resources and practices. So, with the increased gross savings, tourism can be developed at a sustainable rate. These results are also in line with Grilli et al. (2021), which examines the relationship between gross savings and the tourism development. According to the findings of this study, the increased gross savings enhances the financial ability of the firms engaged in the tourism practices or encouraging tourism practices and, thus, develops internal tourism within the country. The results showed that sustainable rural development has a positive relation to the sustainability of tourism development. These results are in line with Ghoochani et al. (2020), which highlight that in the countries where the policies for the SRD are effectively implemented, the natural resources like greenery, natural phenomena, recreational parks, quality food, and more accommodation facilities increase at large scale, and the tourism industry which is totally based on these natural resources and accommodation facilities can make sustainable progress. These results are also supported by Martínez, Martín, Fernández, and Mogorrón-Guerrero (2019), which states that the sustainable development of rural areas provides additional land, buildings, food and other natural resources, and a large labor force to the tourism firms and assists them in making expansion in their business with better facilities and achieve sustainable tourism development.

### Implications

The present study has both theoretical and empirical implications. The study is a great addition to tourism literature. Tourism development is the main point of the study. The study examines the role of economic conditions such as economic development, foreign direct investment, inflation, exchange rate, energy use, sustainable rural development, and gross savings in tourism development. In the previous literature, considering the significance of tourism, the impacts of economic condition on tourism development have been examined but without the specification of economic conditions and their individual analysis. The present study, which examines the economic conditions, namely economic development, foreign direct investment, inflation, exchange rate, energy use, sustainable rural development, and gross savings in tourism development, is an addition to the literature. It is the first time that someone has tried to explore the sources of tourism development in China with the intention that it will contribute to the country’s sustainable development. The present study also has a great significance in emerging economies like China as it highlights how the tourism industry, which is the source of sustainable development (environmental, social, and economic development), can be grown. This study guides the policymakers in establishing policies related to tourism development using different economic conditions of the country. The study suggests to the government that they must try to develop tourism with effective economic policies which give rise to economic development, foreign direct investment, exchange rate, clean energy use, sustainable rural development, and gross savings. Similarly, the study suggests to the economists that they must encourage economic development, foreign direct investment, inflation, exchange rate, clean energy use, sustainable rural development, and increase in gross savings in tourism development.

### Conclusion

In China, the pollution spreading has long been at its peak; so, there is a threat to the sustainability of the country’s growth because of the lack of resources and health damages in future. But the country has the essence to provide for recreation, enjoyment, and learning in the form of recreational places, cultural events, and historical works and places. And tourism growth is the source of protection of the environment, resources, public well-being, and economic growth. That is why the tourism growth within the country must be made swift. The present study intended to pay attention to sustainability of sustainability of tourism development as a basic need of the hour in China. The study was aimed at analyzing the role of economic development, foreign direct investment, inflation, exchange rate, energy use, sustainable rural development, and gross savings in the sustainability of tourism development. The empirical quantitative information about the economic development, foreign direct investment, inflation, exchange rate, energy use, sustainable rural development, and gross savings and their impacts on sustainability of tourism development were arranged to be acquired from China. The results revealed a positive link between economic development, foreign direct investment, inflation, exchange rate, energy use, sustainable rural development, gross savings, and sustainability of tourism development. The results showed that with the rapid progress of country, the financial strength of the government, individual business enterprises, and individuals gets high. This enhances the firms’ power to execute the strategies and plans for the improvement in the tourism services like infrastructure, accommodation, recreation, and traveling. That is why the increase in economic growth leads the country to bring sustainable development in the tourism industry. With investment from both international sources along with internal ones, the tourism sector’s management may carry out various procedures efficiently, and thus, the tourism industry within the country can make sustainable development. The growth in earnings was earned by tourist firms across various tourism service platforms during inflation. Increased revenues allow businesses to upgrade and extend their tourism facilities, allowing them to expand their services. When the exchange rate rises, tourism services offered to foreigners generate higher profits and provide opportunities for tourism companies to innovate in terms of tourism resources, parks, lodging, transport, and other accessories. The use of clean energy maintains tourists’ health and ensures the availability of natural resources such as food, greenery, parks, mountains, hills, waterfalls, and wildlife, all of which provide opportunities for enjoyment; the tourism industry develops with high sustainability. The results showed that when the rural areas are paid attention and developed at sustainable rate, the tourism industry that is mostly dependent on natural resources can be led towards sustainable development. With the increase in the gross savings, the net transfers in the country are large in number, and the strong financial position of the countrymen or firms increases the tourism services and helps gain sustainable development in tourism industry.

### Limitations

Many limitations are associated with the present study. These limitations can be removed during future research on the sustainability of tourism development. The focus of the present study is only on economic factors like economic development, foreign direct investment, inflation, exchange rate, energy use, sustainable rural development, and gross savings, which affect the sustainability of tourism development. The geographical characteristics, social behaviors, cultural values, and government permission all are significant in the case of accelerating sustainability of tourism development. But, since the present study fails to cover all these factors, the research scope is limited. The researchers have the duty to ponder on the study and, with the addition of other essential factors, present a better study. Economic conditions like economic development, foreign direct investment, inflation, exchange rate, energy use, and gross savings have a different proportion of contribution to the sustainability of tourism development in different time periods. The selection of a single or specific time period for the analysis of the role of economic development, foreign direct investment, inflation, exchange rate, energy use, sustainable rural development, and gross savings in creating sustainability in tourism development could not be reliable. So, the authors in future must examine the role of these factors in the sustainability of tourism development during different periods of time for a more reliable study.

## Data Availability

The data that support the findings of this study are attached.
